# A bibliometrics study on the status quo and hot topics of pathogenesis of psoriasis based on Web of Science

**DOI:** 10.1111/srt.13538

**Published:** 2024-01-04

**Authors:** Yujie Yang, Xuwei Zheng, Haiying Lv, Bin Tang, Yang Bi, Qianqian Luo, Danni Yao, Haiming Chen, Chuanjian Lu

**Affiliations:** ^1^ The Second Clinical College of Guangzhou University of Chinese Medicine Guangzhou China; ^2^ State Key Laboratory of Dampness Syndrome of Chinese Medicine The Second Affiliated Hospital of Guangzhou University of Chinese Medicine (Guangdong Provincial Hospital of Chinese Medicine) Guangzhou China; ^3^ Guangdong Provincial Key Laboratory of Clinical Research on Traditional Chinese Medicine Syndrome Guangzhou China; ^4^ Guangdong Provincial Clinical Medicine Research Center for Chinese Medicine Dermatology Guangzhou China; ^5^ Guangdong‐Hong Kong‐Macau Joint Lab on Chinese Medicine and Immune Disease Research Guangzhou University of Chinese Medicine Guangzhou China

**Keywords:** bibliometrics, CiteSpace, pathogenesis, psoriasis, VOSviewer

## Abstract

**Background:**

Psoriasis is an immune‐mediated chronic inflammatory skin disease. Great progress has been made in the pathogenesis of psoriasis in recent years, but there is no bibliometric study on the pathogenesis of psoriasis. The purpose of this study was to use bibliometrics method to analyze the research overview and hot spots of pathogenesis of psoriasis in recent 10 years, so as to further understand the development trend and frontier of this field.

**Methods:**

The core literatures on the pathogenesis of psoriasis were searched in the Web of Science database, and analyzed by VOSviewer, CiteSpace, and Bibliometrix in terms of the annual publication volume, country, institution, author, journal, keywords, and so on.

**Results:**

A total of 3570 literatures were included. China and the United States were the main research countries in this field, and Rockefeller University was the main research institution. Krueger JG, the author, had the highest number of publications and the greatest influence, and Boehncke (2015) was the most cited local literature. *J INVEST DERMATOL* takes the top spot in terms of the number of Dermatol articles and citation frequency. The main research hotspots in the pathogenesis of psoriasis are as follows: (1) The interaction between innate and adaptive immunity and the related inflammatory loop dominated by Th17 cells and IL‐23/IL‐17 axis are still the key mechanisms of psoriasis; (2) molecular genetic studies represented by Long Non‐Coding RNA (LncRNA); (3) integrated research of multi‐omics techniques represented by gut microbiota; and (4) Exploring the comorbidity mechanism of psoriasis represented by Metabolic Syndrome (MetS).

**Conclusion:**

This study is a summary of the current research status and hot trend of the pathogenesis of psoriasis, which will provide some reference for the scholars studying the pathogenesis of psoriasis.

## 1 INTRODUCTION

In 2014, the World Health Organization defined psoriasis as a chronic, noninfectious, disabling, and refractory disease. It affects more than 60 million people worldwide and imposes a heavy economic burden on individuals and societies.^1^ Breakthroughs in the research of the pathogenesis of psoriasis have brought great benefits to patients with psoriasis, such as the research on the pathogenesis of psoriasis tumor necrosis factor‐α (TNF‐α), interleukin‐17 (IL‐17) and IL‐23 targeted biological agents have played an active role in the treatment of moderate to severe, refractory and special types of psoriasis.^2^ Although some progress has been made in targeted therapy for the known pathogenesis of psoriasis, it still cannot solve the problem of recurrence after drug withdrawal. At the same time, there are phenomena of attenuation of efficacy and various adverse reactions.^3^ Therefore, it is necessary and urgent to study the pathogenesis of psoriasis.

Bibliometrics was first proposed by British intelligence scientist Alan Pritchard in 1969. It is a method to analyze the distribution characteristics, quantitative relations and changing rules of books and documents by using mathematics and statistics.^4^ In recent years, thanks to the availability of computer analysis software and the increase in the number of publications, the research efficiency and accuracy of bibliometrics have been improved, and the results can be presented more intuitively through software visualization, making bibliometrics a widely used research method. and these tools have been widely used in medical fields, such as oncology,^5^ Orthopedics,^6^ Hypertension,^7^ and Dermatology.^8^ Although a few of bibliometric studies on psoriasis have been conducted in recent years, they are either regional studies^9^ or only focused on nail psoriasis,^10^ A comprehensive bibliometric study of global psoriasis research has not been conducted. To fill this knowledge gap, this study aimed to perform a bibliometric analysis of publications of psoriasis over the past 11 years (from 2013 to 2022) to identify major contributors and current research status, and to look forward to the research trends and future development prospects in this field.

## 2 METHODS

### 2.1 Search strategy

**FIGURE 1:**
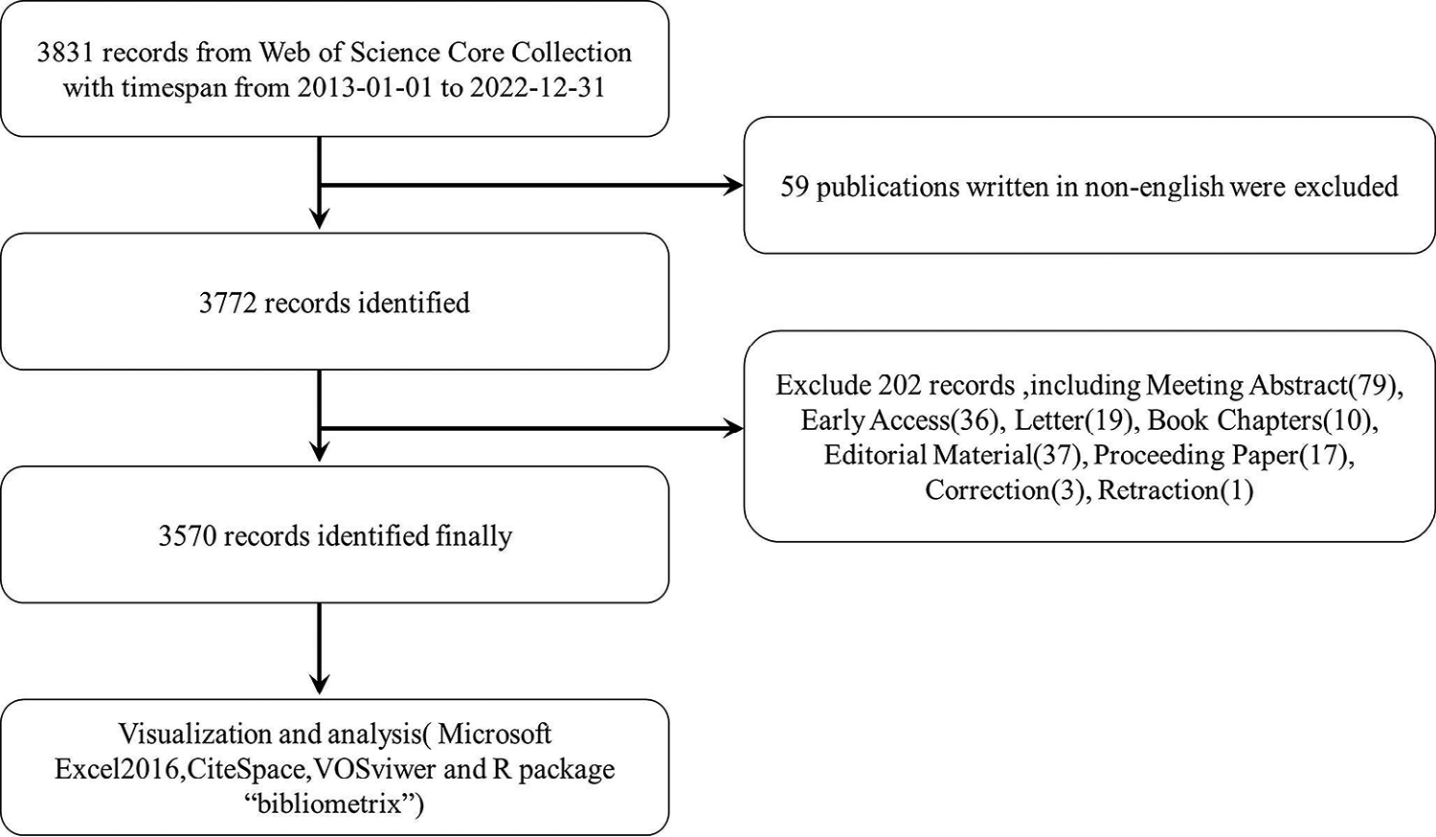
Publications screening flowchart.

We conducted a literature search on the Web of Science Core Collection (WoSCC) database (https://www.webofscience.com/ wos/woscc/basic‐search) on January 14, 2023. The time span was “2013.01.01 to 2022.12.31”. The search formula is ((TS = (Psoriasis)) AND TS = (Pathogenesis)) AND LA = (English), and the type of documents is set to “articles” and “review” (Figure 1).

### 2.2 Data analysis

VOSviewer (version 1.6.18) is a bibliometric analysis software that can extract the key information from numerous publications, which is often used to build collaboration, co‐citation and co‐occurrence networks.^11^, ^12^ In our study, the software mainly completes the following analysis: country and institution analysis, journal and co‐cited journal analysis, author and co‐cited author analysis, and keyword co‐occurrence analysis. In the map produced by VOSviewer, a node represents an item such as country, institution, journal, and author. Node size and color indicate the number and classification of these items, respectively. Line thickness between nodes reflects the degree of collaboration or co‐citation of the items.^13^

CiteSpace (version 6.1.R6) is another software developed by Professors Chen C for bibliometric analysis and visualization.^14^, ^15^ In our study, CiteSpace was applied to map the dual‐map overlay of journals and to analyze reference with Citation Bursts. Parameters were set as follows: Timespan: 2013–2022 (Slice Length = 1), selection Criteria: g‐index (k = 25).

The R package “bibliometrix” (version 3.2.1) (https://www. bibliometrix.org) was applied for a thematic evolution analysis and to construct a global distribution network of publications of psoriasis.^4^, ^11^ The quartile and impact factor of the journal are obtained from Journal Citation Reports 2020. Additionally, Microsoft Office Excel 2019 was used to conduct quantitative analysis of publication.

## 3 RESULTS

### 3.1 Quantitative analysis of publication

**FIGURE 2:**
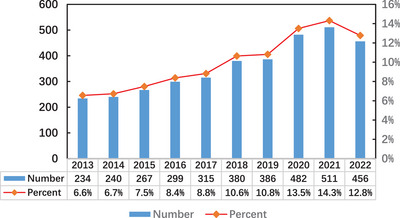
Trends in annual publications of pathogenesis in psoriasis from 2013 to 2022.

According to our search strategy, In the past 10 years, a total of 3570 studies have been published on the pathogenesis of psoriasis, including 2563 articles and 1007 reviews. As shown in Figure 2, the number of publications related to the pathogenesis of psoriasis showed an increasing trend from 2013 to 2021. From 2013 to 2019, the number of publications related to the pathogenesis of psoriasis increased year by year, and maintained a relatively stable growth rate. After the year 2019, the number of publications increased rapidly. At present, 2021 has the highest number of publications (*n* = 511, 14.3%).

### 3.2 Country and institutional analysis

**FIGURE 3:**
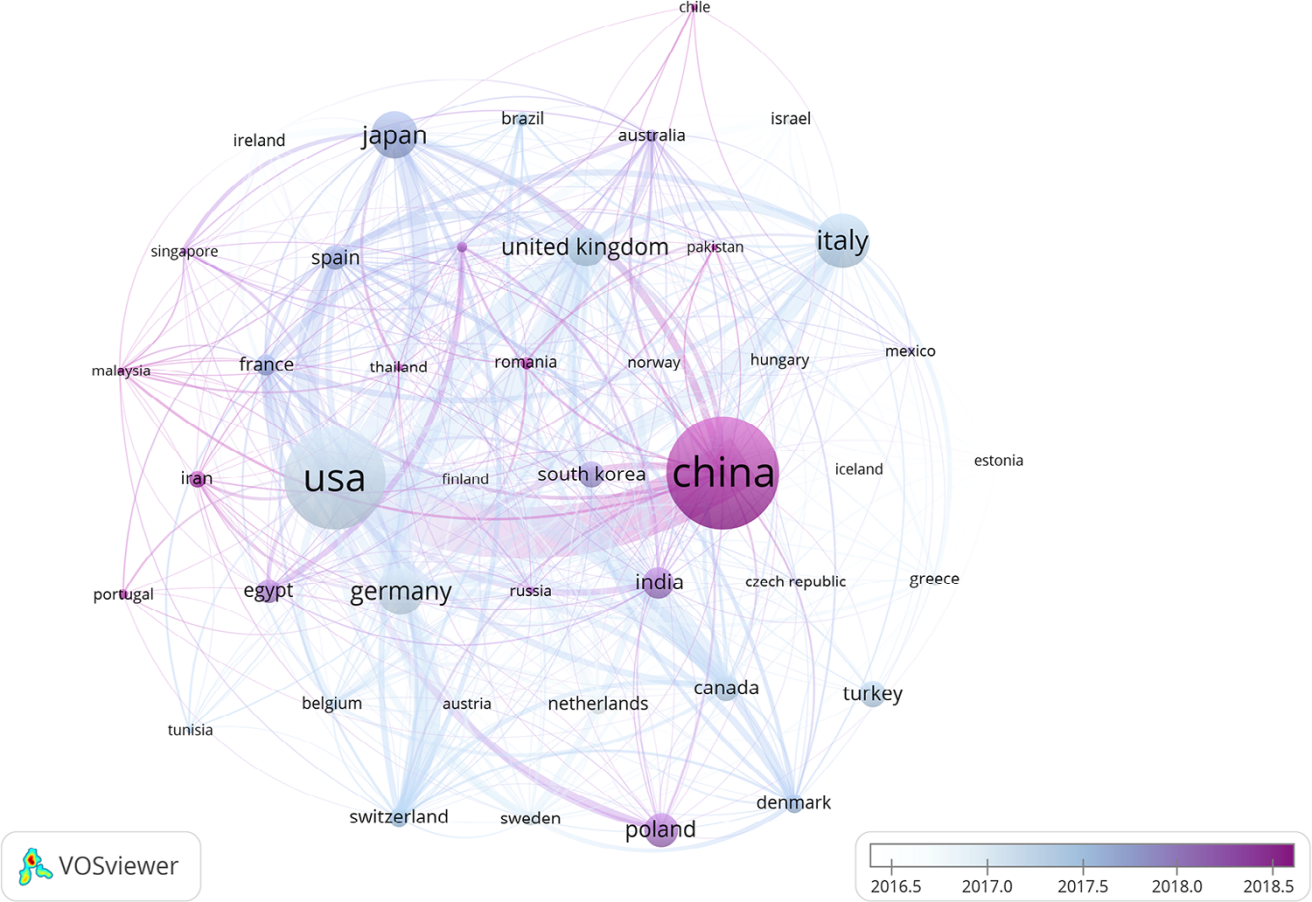
The visualization of country on research of pathogenesis in psoriasis.

**FIGURE 4:**
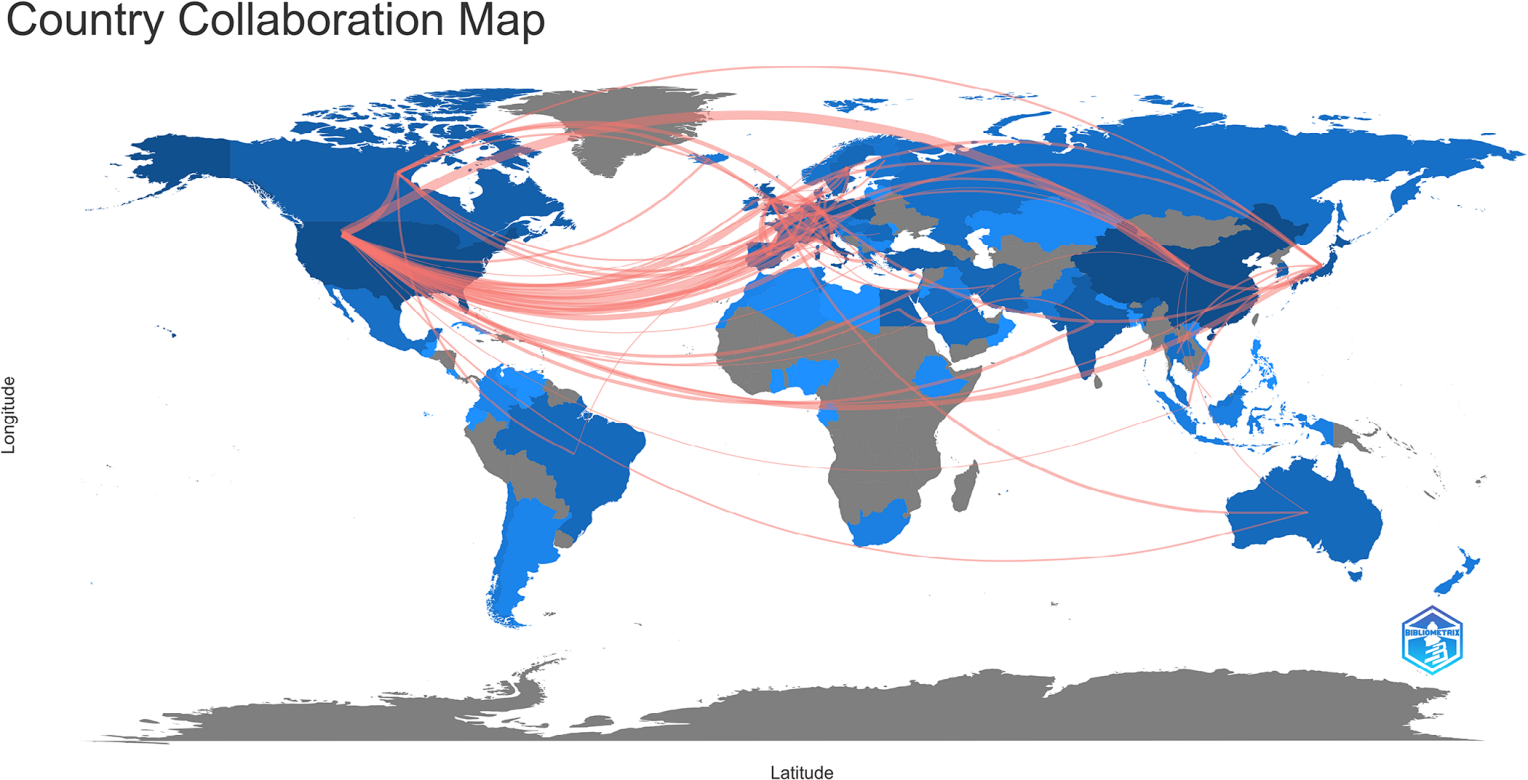
Country collaboration map on research of pathogenesis in psoriasis.

A total of 93 countries and 3699 institutions have participated in the research on the pathogenesis of psoriasis, and the top 10 countries and institutions in terms of publication volume are shown in Table 1. China (*n* = 898, 25.15%), the United States (*n* = 689, 19.30%) and Italy (*n* = 308, 8.63%) ranked top three in the world in terms of the number of articles published in this study. In 2018–2019, China surpassed the United States to become the world's largest country in terms of total number of publications, and has remained there ever since. However, the United States is still the country with the most literature citations. We filtered and visualized 43 countries based on the number of publications more than or equal to 10, and constructed a collaborative network based on the number and relationship of publications in each country (Figure 3). The world Cooperation map (Figure 4) shows the cooperation among countries, among which China has active cooperative relations with the United States, Germany, and the United Kingdom, and the United States has close cooperation with the United Kingdom, Germany, and Canada.

**TABLE 1:** Top 10 countries and institutions on research of pathogenesis in psoriasis.

Rank	Country	Counts	Citations	Organization	Counts	Citations
1	China	(25.15%)	15390	Rockefeller Univ (USA)	(1.65%)	4392
2	USA	(19.30%)	28941	Icahn Sch Med Mt Sinai (USA)	(1.34%)	2025
3	Italy	(8.63%)	7771	Anhui Med Univ (China)	(1.29%)	847
4	Japan	(7.28%)	7476	Univ Roma Tor Vergata (Italy)	(1.29%)	1564
5	Germany	(6.78%)	11805	Fudan Univ (China)	(1.26%)	845
6	United Kingdom	(5.24%)	9408	Fourth Mil Med Univ (China)	(1.12%)	990
7	Poland	(4.73%)	2682	Univ Toronto (Canada)	(1.09%)	1019
8	India	(4.40%)	2639	Univ Calif San Francisco (USA)	(1.04%)	1307
9	Canada	(3.50%)	5897	Shanghai Jiao Tong Univ (China)	(1.01%)	991
10	South Korea	(3.45%)	1723	ShanDong Univ (China)	(0.98%)	485

**FIGURE 5:**
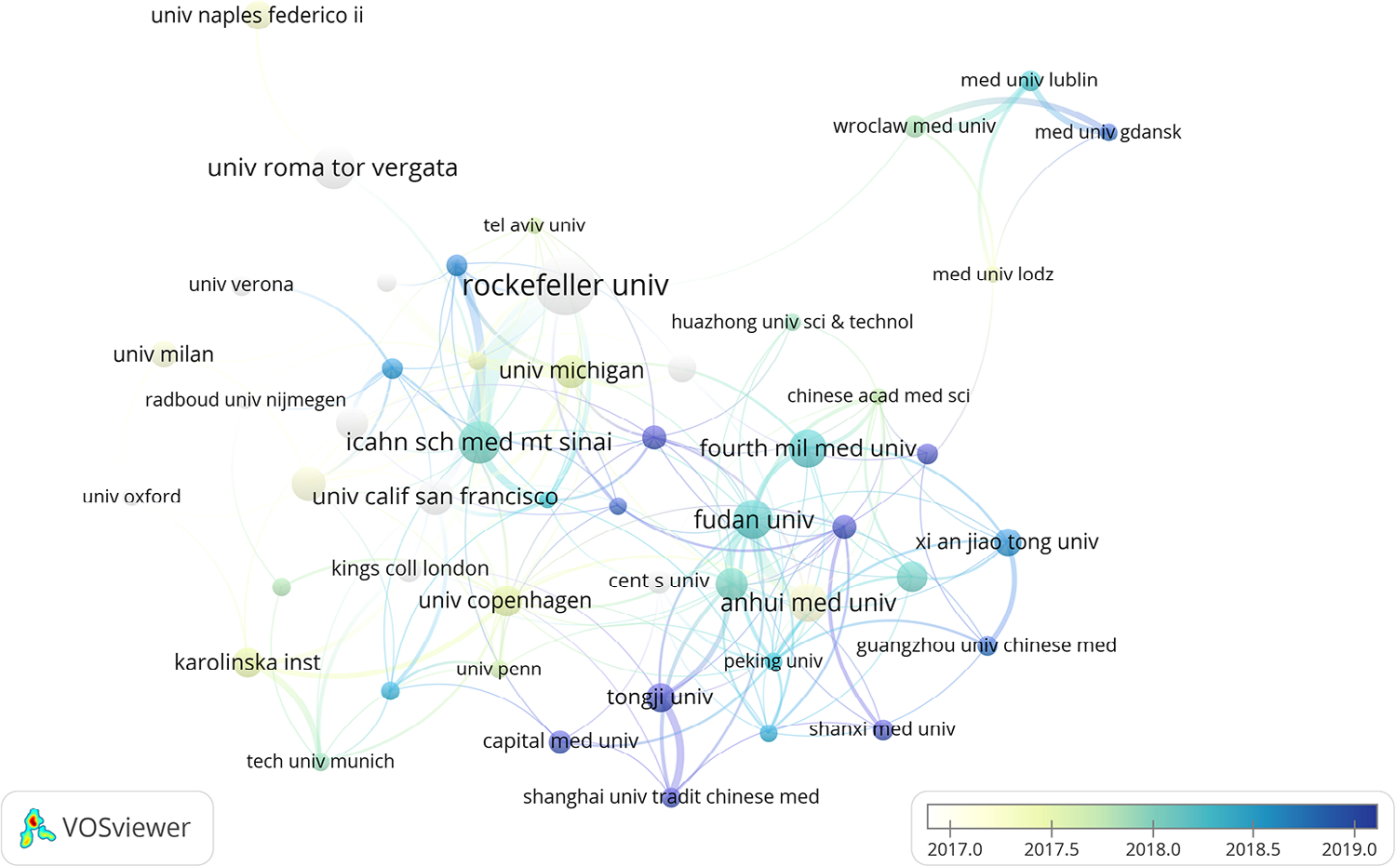
Institutions collaboration map on research of pathogenesis in psoriasis.

We filtered and visualized 48 institutions based on the number of publications more than or equal to 20, and constructed a collaborative network (Figure 5) based on the number and relationship of publications in each institution. Top three institutions were Rockefeller Univ (*n* = 60, 1.65%), Icahn Sch Med Mt Sinai (*n* = 49, 1.35%), Anhui Med Univ and Univ Roma Tor Vergata (*n* = 46, 1.27%). There is close cooperation between the agencies. Rockefeller University has close exchanges with the Icahn School of Medicine at Mount Sinai and the University of Copenhagen. Fudan University has active cooperation with Michigan State University, Copenhagen University and the Fourth Military Medical University.

### 3.3 Authors and co‐cited authors

**FIGURE 6:**
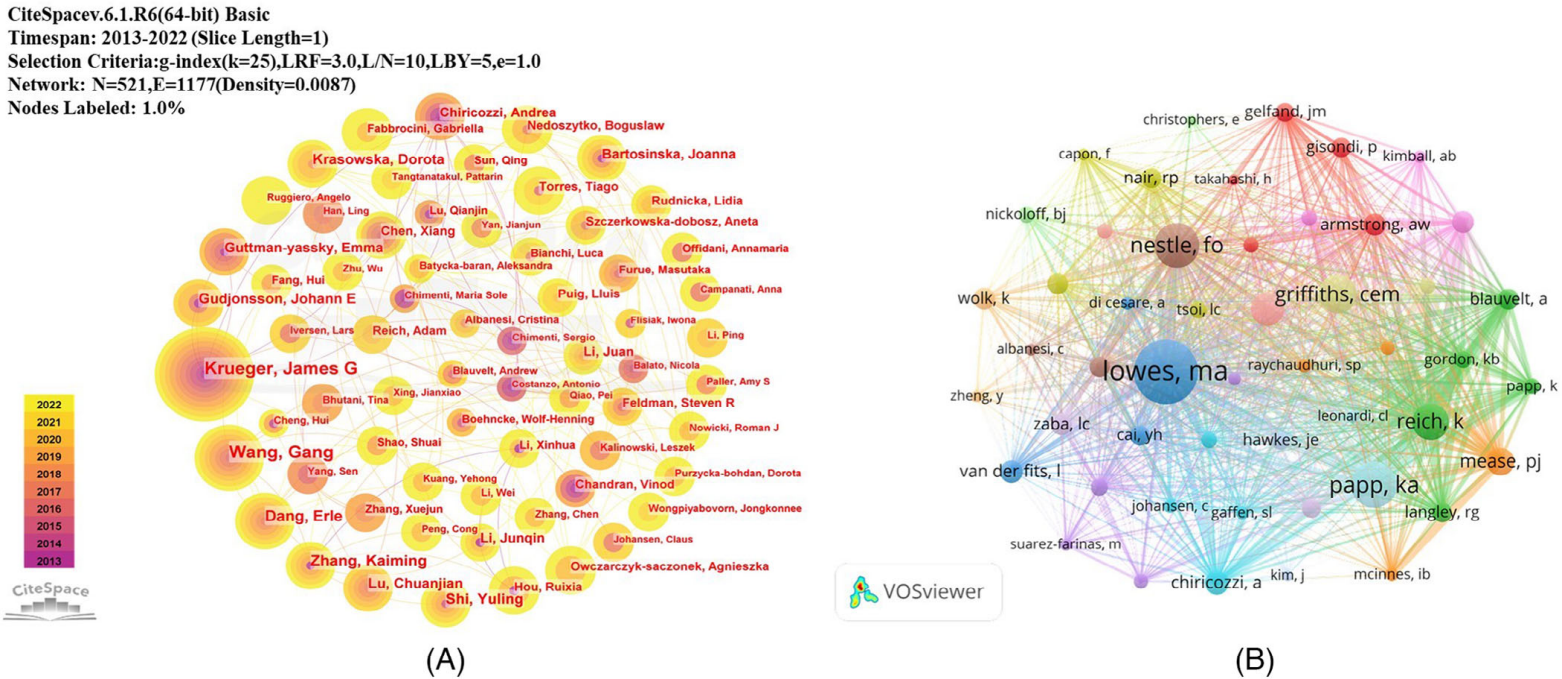
Authors (A) and co‐cited Authors (B) collaboration map.

A total of 1858 authors participated in research on the pathogenesis of psoriasis. The top 10 authors in terms of published papers and co‐cited frequency are shown in Table 2. We build a collaborative network based on authors whose number of published papers is more than or equal to 5 (Figure 6A). The top five scholars in terms of published papers are Krueger JG (*n* = 37, 1.02%), Wang Gang (*n* = 36, 0.99%), Dang Erle (*n* = 23, 0.63%), Zhang Kaiming (*n* = 21, 0.58%), Shi Yuling (*n* = 19, 0.52%). VOSviewer was used to visualize 43 authors whose co‐citation frequency is more than or equal to 200, and construct co‐citation author cooperation network (Figure 6B). Authors with the highest co‐citation frequency are Lowes MA (*n* = 1103), Papp KA (*n* = 781), Nestle FO (*n* = 735), Griffiths CEM (*n* = 667), and Reich K (*n* = 631). H‐index is an index to evaluate the influence of authors, which can reflect the academic achievements of researchers accurately. Of all literatures in this study, the H‐index of Krueger JG, Emma Guttman‐Yassky, and Wang Gang, the top three influential authors, was 27, 19, and 19, respectively.

**TABLE 2:** Top 10 authors and co‐cited authors on research of pathogenesis in psoriasis.

Rank	Author	Counts	Co‐cited author	Citations
1	Krueger, James G	37 (1.02%)	Lowes, MA	1090
2	Wang, Gang	36 (0.99%)	Papp, KA	762
3	Dang, Erle	23 (0.63%)	Nestle, FO	730
4	Zhang, Kaiming	21 (0.58%)	Griffiths, CEM	667
5	Shi, Yuling	19 (0.52%)	Reich, K	631
6	Krasowska, Dorota	17 (0.47%)	Boehncke, WH	608
7	Gudjonsson, Johann E	17 (0.47%)	Mease, PJ	501
8	Lu, Chuanjian	16 (0.44%)	Chiricozzi, A	424
9	Guttman‐yassky, Emma	15 (0.41%)	Van Der Fits, L	417
10	Li, Junqin	14 (0.39%)	Armstrong, AW	414

### 3.4 Journals and co‐cited journals

The top 10 journals in terms of publication and citation are shown in Table 3. Publications related to pathogenesis of psoriasis were published in 812 journals. *Journal of Investigative Dermatology* published most papers (*n* = 113, 3.16%), followed by *International Journal of Molecular Sciences* (*n* = 108, 3.02%), *Experimental Dermatology* (*n* = 102, 2.85%). Among the 812 journals, the journal with the highest impact factor is British Journal of Dermatology (IF: 11.11), followed by *Journal of the European Academy of Dermatology and Venereolo*gy (IF:9.23), *Frontiers in Immunology* (IF:8.79). A total of 67 journals were cited more than 500 times, with the *Journal of Investigative Dermatology* having the highest number of citations at 10 477.

**TABLE 3:** Top 10 journals and co‐cited journals for research of pathogenesis in psoriasis.

Rank	Journals	IF (2022)	Counts	Co‐cited Journal	Citations
1	*J INVEST DERMATOL*	7.59	113	*J INVEST DERMATOL*	10477
2	*INT J MOL SCI*	6.21	108	*BRIT J DERMATOL*	7472
3	*EXP DERMATOL*	4.51	102	*J IMMUNOL*	6914
4	*FRONT IMMUNOL*	8.79	101	*J AM ACAD DERMATOL*	5351
5	*BRIT J DERMATOL*	11.11	69	*ANN RHEUM DIS*	3384
6	*J DERMATOL SCI*	5.41	68	*J EUR ACAD DERMATOL*	3368
7	*J EUR ACAD DERMATOL*	9.23	59	*NEW ENGL J MED*	3345
8	*J DERMATOL*	3.47	54	*PLOS ONE*	3070
9	*ARCH DERMATOL RES*	3.03	51	*J Allergy Clin Immun*	3065
10	*POSTEP DERM ALERGOL*	1.66	50	*NATURE*	2935

**FIGURE 7:**
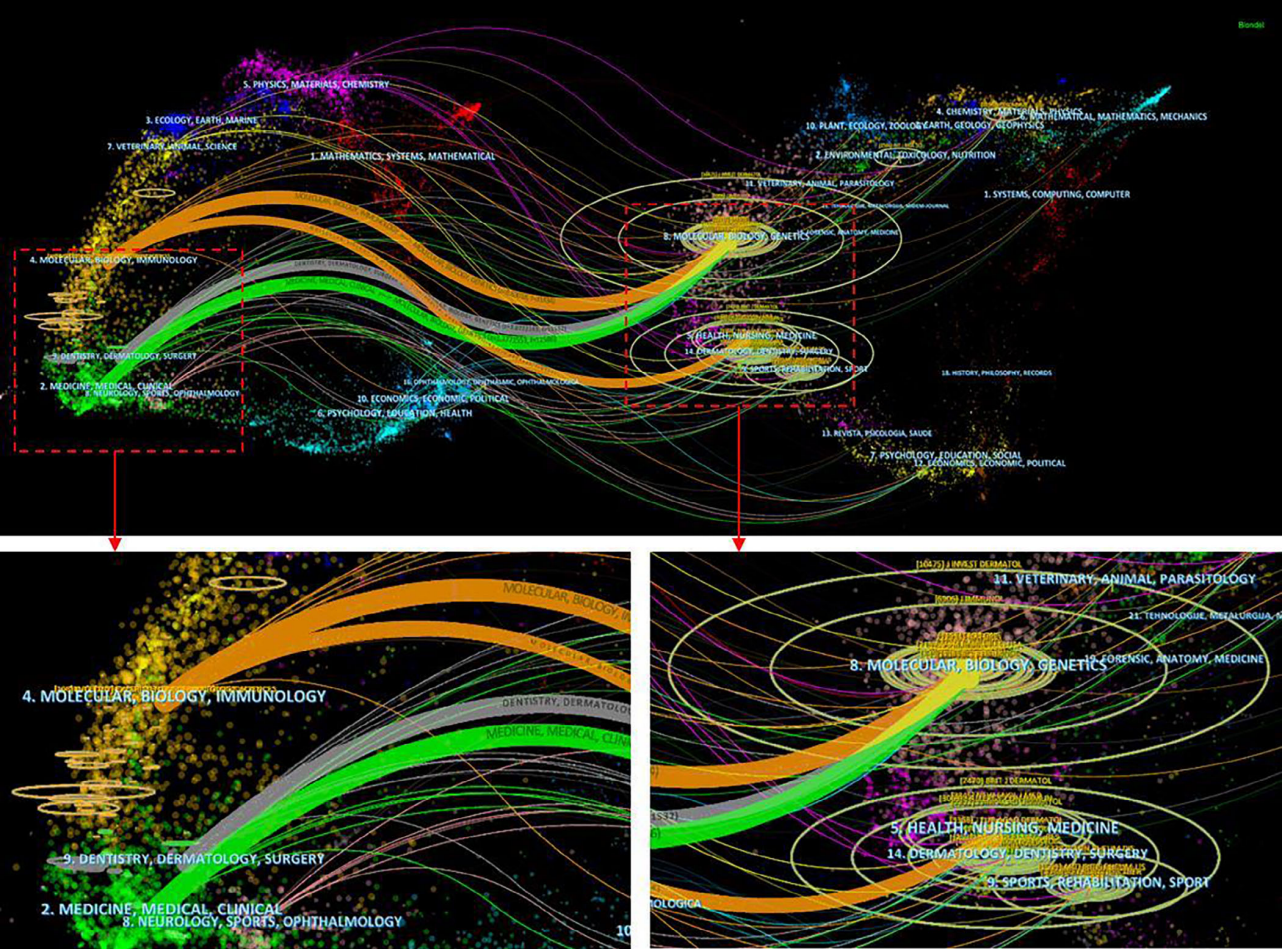
The dual‐map overlay of journals on research of pathogenesis in psoriasis.

The dual‐map overlay of journals (Figure 7) shows the citation relationships between journals and co‐cited journals, with clusters of citing journals on the left, clusters of cited journals on the right and the orange path represents the primary citation path. The three streams of information from different origins covered the fields of molecular biology, immunology, dermatology, surgery, medicine, and clinical medicine, and converged in the fields of molecular, biology, and genetics. It indicated that the study of molecular interactions from the perspective of genetics may be the current research hotspot and trend in elucidating the pathogenesis of psoriasis.

### 3.5 Co‐cited references

There are 3570 co‐cited references on research of pathogenesis of psoriasis over the past 10 years. In top 10 co‐cited references (Table 4), all references were co‐cited over 100 times, and the top one reference Boehncke (2015) was co‐cited more than 300 times. Among them, eight were reviews and two were articles, all of which were important in this field.

**TABLE 4:** Top 10 local cited references on research of pathogenesis in psoriasis.

Author (Year)	local cited references	Local citations
Boehncke, 2015^64^	Psoriasis	363
Langley, 2014^65^	Secukinumab in Plaque Psoriasis — Results of Two Phase 3 Trials	208
Hawkes, 2017^66^	Psoriasis pathogenesis and the development of novel targeted immune therapies	194
Rendon, 2019^67^	Psoriasis Pathogenesis and Treatment	146
Lowes, 2013^68^	The IL‐23/T17 pathogenic axis in psoriasis is amplified by keratinocyte responses	129
Lande, 2014^69^	The antimicrobial peptide LL37 is a T‐cell autoantigen in psoriasis	127
Martin, 2013^70^	The emerging role of IL‐17 in the pathogenesis of psoriasis: Preclinical and clinical findings	126
Harden, 2015^71^	The immunogenetics of Psoriasis: A comprehensive review	125
Armstrong, 2020^72^	Pathophysiology, Clinical Presentation, and Treatment of Psoriasis	107
Takeshita, 2017^73^	Psoriasis and comorbid diseases: Epidemiology	105

### 3.6 Keywords and hotspots analysis

**FIGURE 8:**
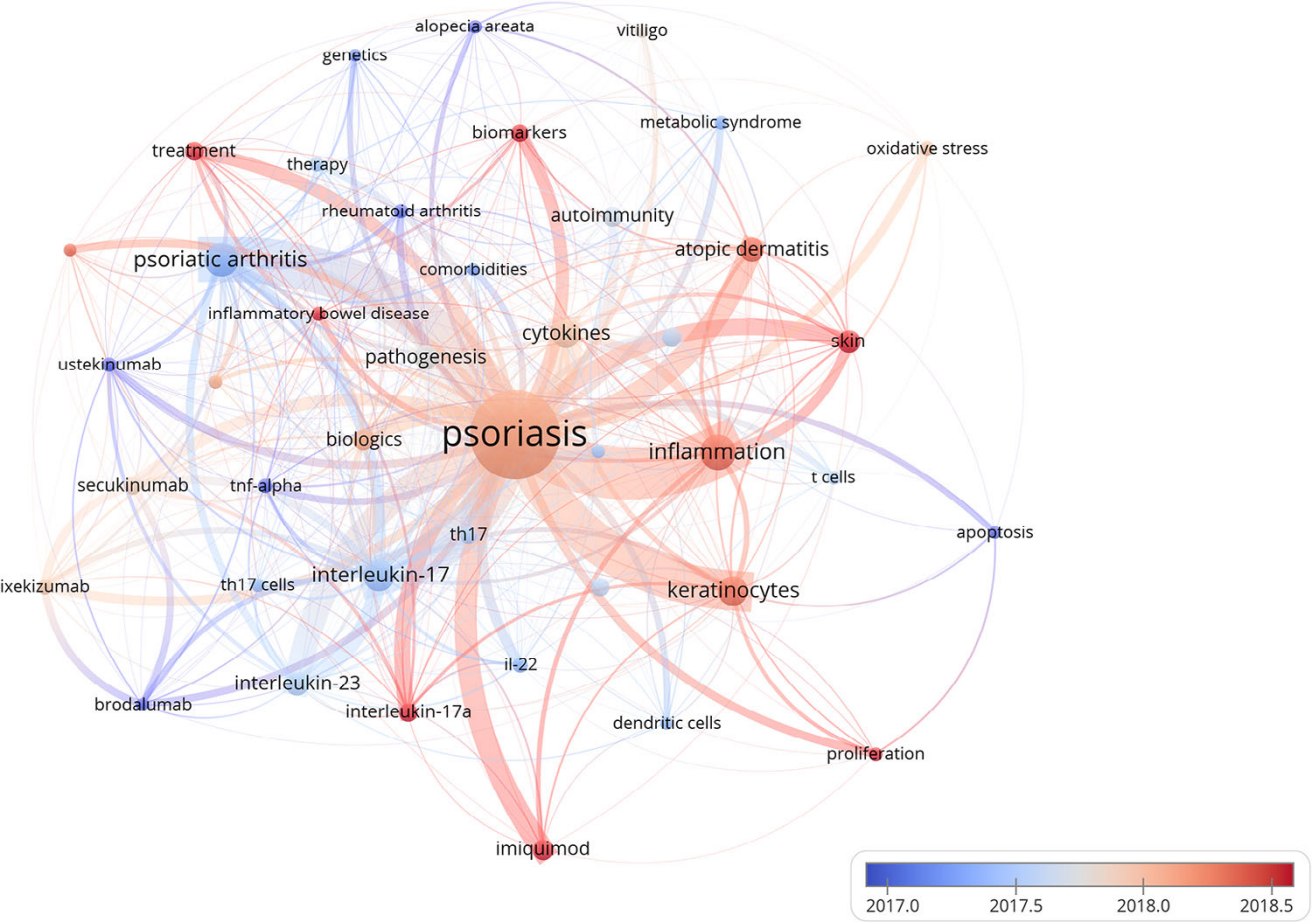
The visualization of keywords on research of pathogenesis in psoriasis.

Through the co‐occurrence analysis of keywords, we could quickly capture research hotspots in a certain field. A total of 10387 keywords were retrieved in this study, and 43 keywords with a keyword occurrence frequency more than or equal to 100 were screened and visualized to construct network (Figure 8). The larger the node, the higher the frequency of keyword occurrence. The line width between nodes represents the degree of correlation between keywords. The figure shows that the high‐frequency keywords related to the pathogenesis of psoriasis involve “inflammation”, “cytokines”, “IL‐17”, “IL‐23”, “biological agents”, “biomarkers”, “keratinocytes”, “oxidative stress”, “proliferation”, “apoptosis”, and so on. Table 5 shows the 20 keywords with the highest frequency.

**TABLE 5:** Top 20 keywords on research of pathogenesis in psoriasis.

Rank	Keyword	Counts	Rank	Keyword	Counts
1	psoriasis	2258	11	double‐blind	321
2	pathogenesis	1149	12	il‐17	309
3	inflammation	782	13	t‐cells	305
4	expression	776	14	th17 cells	270
5	skin	553	15	rheumatoid‐arthritis	263
6	cytokines	435	16	activation	259
7	keratinocytes	392	17	therapy	253
8	psoriatic arthritis	391	18	tnf‐alpha	249
9	disease	376	19	dendritic cells	217
10	cells	344	20	differentiation	216

**FIGURE 9:**
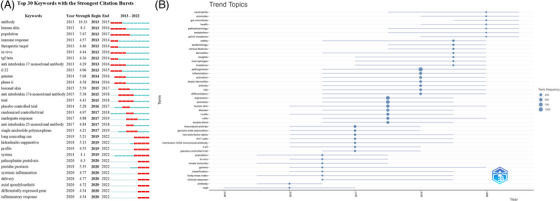
Keywords with strong citation bursts (A) and trend topics analysis (B).

The emergence and trend of keywords can track the trend of researchers in the pathogenesis of psoriasis, and help to quickly capture the research hotspot in this field. The emergence analysis results of keywords in core English literature in recent 10 years are shown in Figure 9(A), in which the red bar chart indicates strong keyword emergence, indicating that the keyword has emerged intensively in the corresponding years. As shown in the figure, anti‐interleukin 17a monoclonal antibody, immune response and TGF‐beta attracted much attention from scholars during 2013–2015. From 2016 to 2018, There are more studies in the field of single nucleotide polymorphism, anti‐interleukin 23 monoclonal antibody and randomized controlled trial. In recent years (2019–2022), long noncoding RNA, systemic inflammation, differentially expressed gene, pustular psoriasis, and inflammatory response have become the focus of research on the pathogenesis of psoriasis. The results of trend topic map showed that the topics related to this field in recent years included neutrophil, gut microbiota, metabolism, ap1s3 mutations, etc. (Figure 9B).

## 4 DISCUSSION

### 4.1 General information

Research on the pathogenesis of psoriasis began in the 1940s, but the number of articles published at that time was limited. From 1940 to 1990, the cumulative number of articles was less than 20. From 1991 to 2006, the number of papers published gradually increased, with an annual average of 51 papers. Since 2007, the number of papers published in this field has exceeded 100 annually. In the past 10 years, the number of relevant papers has increased rapidly, indicating that the research on the pathogenesis of psoriasis is in a period of explosion, and related studies have attracted more and more scholars' attention.

China and the United States are major countries conducting research on the pathogenesis of psoriasis, and China ranks first. About 50% of the top 10 research institutions are located in China, followed by the United States (*n* = 3, 30%). We noticed the close cooperation among four countries: the United States, China, United Kingdom, and Germany. When it comes to research institutions, there is a good cooperative relationship between some of them, such as Rockefeller University, Icahn School of Medicine at Mount Sinai, and University of Rome II‐Tor Vergata. However, we also found some institutions, such as SiChuan University, Cairo University, had little collaboration with other institutions, which will be detrimental to the long‐term development of academic research. We strongly recommend research institutions in various countries carry out extensive cooperation and communication to jointly promote the development of the pathogenesis of psoriasis.

Most of the research on the pathogenesis of psoriasis was published in *Journal of Investigative Dermatology* (IF = 7.59, Q1), indicating it is currently the most popular journal in this research field. Among the journals, the journal with the highest impact factor is *British Journal of Dermatology* (IF = 11.11, Q1), followed by *Journal of European Academy of Dermatology and Venereology* (IF = 9.23, Q1). Regarding the co‐cited journals, we could find most of them are high‐impact Q1 journals. Obviously, these journals are high‐quality international journals, providing support for the study on the pathogenesis of psoriasis. What's more, the current research of pathogenesis of psoriasis is mainly published in molecular, immunology and clinically related journals, indicating that basic research and clinical application in this field are closely combined. Effective transformation the basic research results of pathogenesis of psoriasis into clinical application will bring huge benefits to patients.

From the perspective of the author, Krueger JG, Wang Gang, Dang Erle, Zhang Kaiming and Shi Yuling published the most articles. According to the H‐index, Krueger JG, Emma Guttman‐Yassky, and Wang Gang are the top three influential authors. Professor Krueger JG has in‐depth research on the pathogenesis of psoriasis, which includes the driving effects of cytokines other than IL‐17A in the IL‐17 family on the inflammatory pathways associated with psoriasis^16^; inflammatory loops^17^; the imbalance between Th17 cell subtypes (e.g., regulatory dendritic cells, type 1 Tregs) and regulatory immune cell subsets in psoriasis; small molecule drugs (tyrosinase 2 inhibitor) and so on.^18^, ^19^, ^20^ Professor Wang Gang mainly studied psoriasis, immunological diseases and various skin diseases. His research on the pathogenesis of psoriasis has made significant contributions to Langerhans cells, inflammatory responses mediated by inflammasomes like Nod‐like receptor pyrin domain containing 3 (NLRP3) and Absent in melanoma 2 (AIM2)^21^, ^22^; the polarization effect of miR‐381‐3p on Th1 and Th17^23^; the differentiation effect of glycolysis metabolism on Th17 and Th1^24^; adipocytes and C1q/tumor necrosis factor‐related protein 3 (CTRP3) mediated Lysosome‐associated membrane protein 1 (LAMP1)/ Signal transducers and activators of transcription (STAT3) axis^25^ in psoriasis. Professor Emma Guttman‐Yassky focuses on psoriasis and atopic dermatitis, believing that although there are important clinical and mechanism differences between the two diseases, they also share common pathogenesis, including infiltration of circulating immune cells in the skin, changes in the expression of pro‐inflammatory cytokines, and skin barrier damage.^26^ In her research on the pathogenesis of psoriasis, she focuses on the pro‐inflammatory mechanisms of CD14+3 dendritic cells in psoriasis^27^; the synergistic induction of psoriatic gene expression responses by IL‐36 and IL‐17A in keratinocytes^28^; and regulatory immune cell subsets.^18^ Interestingly, we noticed that of all the top 10 influential authors, Lu Chuanjian is the only one who focus on alternative medicine (Chinese medicine). With the development of alternative medicine, more and more attentions have been payed on the pharmacological mechanism of phytochemicals. Phytochemicals have a huge research space in the field of psoriasis treatment, which is worthy of in‐depth research by scholars from all over the world in the future.

### 4.2 Hotspots and frontiers

This study reviewed the current status of research on the pathogenesis of psoriasis, and the results showed that the interplay between the innate and adaptive immune systems, Th17 cells and IL‐23/IL‐17 axis related cells and inflammatory factors, and genetic factors are still the key mechanisms of psoriasis. The research hotspots and development trends in this field reflected by keywords showed that immune factors and inflammatory response were still the core of this field; Molecular biology research represented by long non‐coding RNA has a broad research space in this field; multi‐omics technology represented by gut microbiota has broad research prospects in this field; The comorbidity mechanism of psoriasis represented by metabolic syndrome is one of the focuses of researchers.

#### 4.2.1 Long non‐coding RNAs

The rise of high‐throughput technologies has allowed researchers to conduct a comprehensive and detailed analysis of the human genome and transcriptome, and many non‐coding RNAs associated with psoriasis susceptibility genes have been identified. The first is the research on miRNA (microRNA). With the gradual deepening of miRNA research and driven by bioinformatics and high‐throughput sequencing technology, the veil of lncRNA and circRNA (circular RNA) has been gradually revealed. lncRNA is a non‐coding RNA with a length of more than 200 nucleotides. It can play an important role in epigenetic, transcriptional, and post‐transcriptional gene expression by regulating target genes.^29^ circRNA is a kind of circular long non‐coding RNA, which can be classified as a subclass of lncRNA.

lncRNAs are involved in the pathogenesis of psoriasis mainly through the following pathways: (1) lncRNAs can directly participate in gene regulation. PRINS, an RNA gene associated with susceptibility to psoriasis induced by stress, is a primate specific LncRNA.^30^ G1P3 is an anti‐apoptotic gene regulated by PRINS. Compared with normal skin tissues, GIP3 gene regulated by PRINS was up‐regulated by 400 times in psoriatic lesions and nine times in non‐skin lesions.^31^ Vitro experiments showed that downregulation of G1P3 inhibited spontaneous apoptosis of keratinocytes. PRINS may be involved in the pathogenesis of psoriasis by altering the expression of G1P3 and reducing the sensitivity of keratinocytes to spontaneous apoptosis.^30^, ^31^ (2) LncRNA can participate in the regulation through the mechanism of competing endogenous RNA (ceRNA), that is, LncRNA can act as a molecular sponge for miRNA, adsorb miRNA and regulate the expression of miRNA target genes. Xian J et al. found that LncRNA AGAP2‐AS1 promotes keratinocyte proliferation and inhibits apoptosis. m6A methyltransferase METTL3‐mediated AGAP2‐AS1 acts as a ceRNA to up‐regulate AKT3, activate AKT/mTOR pathway and promote keratinocyte proliferation by adsorb miR‐424‐5p.^32^ The results of Lin J et al. suggest that LncRNA regulate the expression of genes in the JAK/STAT signaling pathway by competing for miR‐545‐5p and promote the inflammatory response in psoriasis.^29^ (3) LncRNAs are involved in the regulation of immune and inflammatory responses. A number of studies have shown that dysregulation of LncRNA expression in psoriasis plays an important role in the pathogenesis of psoriasis by affecting cell function and activity and inducing immune inflammatory response. LncRNA SPRR2C is highly expressed in psoriatic lesions and HaCaT cells, and the expression of SPRR2C in tissue samples is positively correlated with the severity of psoriatic lesions. Knockdown of SPRR2C inhibited IL‐22‐induced proliferation of HaCaT and decreased the mRNA expression of inflammatory factors IL‐1β, IL‐6, and TNF‐α.^33^ LncRNA‐MSX2P1, MIR31HG, and lncRNA‐H19 are up‐regulated in psoriasis lesions, which can affect the proliferation and apoptosis of keratinocytes and induce the production of inflammatory factors.^34^, ^35^, ^36^ lncRNA FLICR inhibits Treg cell activity by down‐regulating Foxp3 expression.^37^ LncRNA MALAT1 is related to the tolerogenic function of dendritic cells and the induction of immune tolerance.^38^

#### 4.2.2 Gut microbiota

The development of high‐throughput sequencing technology and the enrichment of omics database and algorithm tools have promoted the rapid development of multi‐omics. The application of multiple omics technologies, such as genomics, transcriptomics, proteomics, and metabolomics, enables researchers to analyze biological physiological functions and pathological mechanisms of diseases at the microscopic level, and the biological information hidden behind big data greatly expands the cognitive horizon of researchers.

Gut microbiota plays an important role in maintaining the body's autoimmune homeostasis. The imbalance of gut microbiota can have a direct impact on intestinal immune cells such as T cell subsets, neutrophils, natural killer lymphocytes, and macrophages.^39^ More and more evidence has shown that the pathogenesis of psoriasis is related to the disorder of gut microbiota. The number, abundance and proportion of gut microbiota in patients with psoriasis are significantly changed.^40^, ^41^ The disorder of gut microbiota may induce the activation of systemic inflammation by affecting the function of immune cells and the secretion of cytokines, and affect the development of psoriasis.^42^ In addition to immune‐inflammatory pathways, the involvement of gut microbiota disorders in the pathogenesis of psoriasis may be related to the following factors: (1) Intestinal barrier function. Gut microbiota can not only closely combine with intestinal mucosal cells to form a biological mechanical barrier of the intestine, but also form a chemical barrier with the mucus secreted by intestinal epithelial cells to maintain the autoimmune homeostasis of the human body.^43^ The disorder of gut microbiota can destroy the intestinal mucosal barrier, increase the permeability of intestinal mucosa, and aggravate the inflammatory response of psoriasis.^44^ (2) Metabolites. The decrease of intestinal dominant bacteria in patients with psoriasis can affect the secretion of Short chain fatty acids (SCFAs) and Medium‐chain fatty acids (MCFAs). SCFAs and MCFAS play an irreplaceable role in the regulation of Th1/Th2, Th17/Treg and other immune balance, and are also related to cell energy, nutrient metabolism, physical barrier, etc.^42^, ^45^ Faecalibacterium prowazekii, an intestinal probiotic, is an important source of SCFA butyrate. Butyrate can provide energy for colon cells, reduce oxidative stress, and play an anti‐inflammatory role by triggering regulatory T cells, thus giving immune tolerance outside the gastrointestinal system. Butyrate is also involved in the regulation of various inflammatory factors, such as TNF‐α, IL‐10, IL‐1β and so on. However, compared with healthy people, this strain is significantly reduced in the gut of patients with psoriasis.^39^, ^46^

#### 4.2.3 Metabolic syndrome

Patients with psoriasis are often complicated with psoriatic arthritis, cardiovascular disease, inflammatory bowel disease, and metabolic syndrome. Therefore, psoriasis is generally considered to be a systemic inflammatory disease. In patients with psoriasis, the prevalence of MetS ranges from 20% to 50% and increases with the severity of psoriasis.^47^ A large population‐based study in the United Kingdom found that patients with psoriasis had a higher prevalence of MetS compared with controls (OR: 1.50, 95%CI: 1.40–1.61). Patients with mild, moderate, and severe psoriasis had 22%, 56%, and 98% increased odds of MetS relative to controls, respectively.^48^

Psoriasis and MetS share common pathogenesis: (1) Cytokines. Psoriasis and MetS share common pathogenic factors, such as adipokines adiponectin, leptin, retinol‐binding protein‐4, lipocalin‐2, and pro‐inflammatory cytokines TNF‐α, IL‐17A, IL‐1β, and IL‐6.^49^ Altered levels of related cytokines may contribute to metabolic disorders and the development of psoriatic skin inflammation. Leptin is a key hormonal regulator of metabolism. Elevated levels of leptin are observed in obese individuals and patients with psoriasis, and are positively correlated with the severity of psoriasis, and can be reduced by acitretin, a systemic anti‐inflammatory drug for psoriasis.^50^ Adiponectin is an anti‐inflammatory adipokine whose levels are reduced in metabolic disorders, obesity, and type 2 diabetes.^51^ Because adiponectin is associated with the severity and incidence of MetS, adiponectin may serve as a predictive marker of MetS.^52^ Studies have shown that adiponectin can inhibit the synthesis of IL‐17 and regulate skin inflammation, which may be a target for the treatment of psoriasis.^53^ In addition, psoriasis and MetS share a common state of chronic low‐grade inflammation, and inflammatory markers such as IL‐6 and TNF‐α are increased in patients with psoriasis and MetS.^54^ The common disorders of adipokine and cytokine secretion may be a bridge linking psoriasis and MetS. (2) Oxidative stress and Endoplasmic Reticulum (ER) stress. Both psoriasis and MetS patients have lipid, DNA, and protein oxidative damage.^55^, ^56^ The imbalance of oxidative stress in psoriasis can activate signaling pathways such as nuclear factor kappa‐B (NF‐κB) and mitogen activated protein kinase (MAPK). It leads to the activation of Th1 and Th17 cells and the secretion of proinflammatory cytokines, which leads to the excessive proliferation of keratinocytes, immune cell infiltration, and changes in vascular permeability through lipid peroxides.^57^ Oxidative stress‐targeted therapy may be a potential preventive strategy for psoriasis and MetS by regulating oxidative stress. Besides, the overexpression of ER stress‐related proteins, including binding immunoglobulin heavy chain protein and C/EBP homologous protein, in the epidermis of patients with psoriasis suggests increased ER stress in keratinocytes from patients with psoriasis. In addition, psoriasis related proinflammatory mediators such as TNF‐α, IL‐1β, IL‐17A, and IFN‐γ can induce endoplasmic reticulum stress in a variety of immune cells.^58^, ^59^, ^60^, ^61^ Long‐term endoplasmic reticulum stress is also a key factor in the pathogenesis of metabolic syndrome. The application of ER stress inhibitors can improve metabolic parameters and reduce mets‐induced cardiovascular complications in rats with MetS.^62^ (3) Gut microbiota imbalance. The damage of intestinal barrier caused by the disorder of gut microbiota can cause bacterial translocation, produce endotoxins or harmful metabolites into the circulation system, and then cause systemic inflammation and aggravate MetS and psoriasis.^49^, ^63^

## 5 ADVANTAGES AND SHORTCOMINGS

Although Web of Science is the most commonly used database for bibliometric measurement, with a relatively large number of literatures included and high literature quality in its core data set, this study still has a single data source and few literatures included, which may omit literatures not included in this database. In addition, the analysis period of this study is limited to the past 10 years, which cannot reflect the earlier research changes in this field. Finally, the application of bibliometrics can better reflect the past research hotspots in this field, but it is not enough to present the emerging research hotspots and cannot accurately predict the future research trend.

## 6 CONCLUSIONS

With bibliometric method, we found that the pathogenesis of psoriasis has attracted increasing attention in the past decade, and institutions and scholars in China and the United States have made outstanding contributions to the development of this field. Immune‐mediated inflammatory response is still a key mechanism in the field of psoriasis. There is a broad research space for molecular biology research and multi‐omics integrated analysis centered on immune inflammation. Among them, the role of long non‐coding RNA and gut microbiota in the pathogenesis of psoriasis has attracted much attention. At present, psoriasis is considered as a systemic inflammatory disease, and the in‐depth study on the comorbidity of psoriasis may be a breakthrough to discover the potential pathogenesis, which is increasingly favored by researchers.

## CONFLICT OF INTEREST STATEMENT

The authors declare that the research was conducted in the absence of any commercial or financial relationships that could be construed as a potential conflict of interest.

## Data Availability

The original contributions presented in the study are included in the article/supplementary material. Further inquiries can be directed to the corresponding author.
